# Unmet Care Needs among Veterans: Intersection of Homebound Status and Rurality

**DOI:** 10.1093/geroni/igaf122.4040

**Published:** 2025-12-31

**Authors:** Sandra Garcia-Davis, Way Way Hlaing, Denise Vidot, Daniel Feaster, Erin Bouldin, Ranak Trivedi, Luci Leykum, Stuti Dang

**Affiliations:** Nova Southeastern University, Fort Lauderdale, Florida, United States; University of Miami Division of Epidemiology & Population Health Sciences, Miami, Florida, United States; School of Nursing and Health Studies, Coral Gables, Florida, United States; University of Miami Miller School of Medicine, Miami, Florida, United States; University of Utah, Salt Lake City, Utah, United States; VA Palo Alto Health Care System, Palo Alto, California, United States; South Texas Veterans Healthcare System, San Antonio, Texas, United States; Miami VA Healthcare System, Miami VA Healthcare System, Florida, United States

## Abstract

Most older adults prefer aging in place, even when homebound. Equitable access to long-term services, including home and community-based services (HCBS), is a Veterans Affairs (VA) priority, yet rural Veterans often face access challenges. Here, we describe the prevalence of activities of daily living (ADL) and instrumental ADL (IADL) unmet needs by homebound status and rurality and assess the association between homebound status and unmet needs, stratified by rurality. We analyzed Veterans aged 65+ with ADL or IADL problems from the HERO CARE survey. Veterans reporting difficulty with at least one ADL or IADL were categorized as having a problem. Unmet need was defined as requiring “a little or a lot more help.” Weighted multivariable modified Poisson regressions with robust standard errors assessed the association between homebound status and unmet need, stratified by rurality. Among Veterans with ADL or IADL problems (N = 5,045; Weighted N = 190,224), 15% were homebound and 21% rural. About 13% of rural and 15% of urban Veterans were homebound. ADL unmet needs were reported by 10% of rural and 15% of urban Veterans (p<.05). Rural homebound Veterans had 2.01 (1.14–3.43) times higher prevalence of ADL unmet needs than rural non-homebound Veterans. Urban homebound Veterans had 1.45 (1.18–1.85) times higher prevalence than urban non-homebound Veterans. No significant IADL associations were found. Unmet ADL needs vary by homebound status and rurality. There is a critical need to understand factors driving unmet needs and access disparities among homebound rural Veterans, so equity-focused solutions can be developed.

